# Computed Tomography Volumetric Measurements of Adrenal Glands in 26 Dogs Under One Year of Age: A Retrospective Study

**DOI:** 10.3390/vetsci12100974

**Published:** 2025-10-10

**Authors:** Julia Topmöller, Johanna Rieder, Sebastian Meller, Kerstin von Pückler, Holger Volk, Kristina Merhof

**Affiliations:** Department of Small Animal Medicine and Surgery, University of Veterinary Medicine Hannover, 30559 Hannover, Germany

**Keywords:** adrenal glands, computed tomography, imaging

## Abstract

Limited data exist regarding the size and volume of adrenal glands in puppies; therefore, the present research aims to describe volumetric and traditional measurements of adrenal glands in computed tomography (CT) images of 26 dogs under 1 year of age. Using OsiriX^®^MD v9.0.1, the adrenal volume as well as adrenal length, and the height and width of the cranial and caudal poles were documented. The results were compared with groups based on age, weight at the time of examination, and the dogs’ adult size when patients were clinically re-evaluated after more than 12 months of age. The mean adrenal gland volumes were 0.50 cm^3^ for the left (range 0.08–1.29 cm^3^) and 0.41 cm^3^ for the right (range 0.03–1.10 cm^3^) adrenal gland. The results showed that older puppies had larger adrenal glands, although the difference did not reach statistical significance. The volume of the adrenal glands correlated positively with body weight and the patients’ adult size. The findings highlight the diagnostic potential of CT-based adrenal volumetry and two-dimensional measurements and support their use in refining reference values for young dogs. The strong correlation between adrenal size and body weight emphasizes the importance of weight-adjusted interpretation in clinical settings.

## Simple Summary

This study examined adrenal gland size in puppies under one year of age using computed tomography (CT) volumetry and traditional measurements, including length, height, and width. Adrenal volume showed a positive correlation with body weight, highlighting the need for weight-adjusted reference values. Age-related trends and mild asymmetry between the left and right glands were noted. CT proved to be a reliable method for assessing adrenal glands in young dogs; however, larger studies including endocrine data are required to ensure adequate hormonal influences.

## 1. Introduction

Adrenal gland diseases are common in dogs, with hyperadrenocorticism and hypoadrenocorticism being the most prevalent conditions [1,2,3]. Both diseases can cause changes in the size and structure of the glands [4].

The challenge of diagnosing hypoadrenocorticism arises from the variable clinical symptoms and laboratory changes that are nonspecific and can differ among patients. Reference values for laboratory parameters vary naturally with age; for example, the normal leukocyte count differs in juvenile animals compared to adult dogs because of the increased reactivity of their immature immune systems. In contrast to adult dogs, in which neutrophilic granulocytes are the predominant leukocyte type, lymphocytes mainly make up the primary cell population in juvenile dogs. This difference can be especially significant when diagnosing or managing Addison’s disease, making a definitive diagnosis in juvenile dogs even more difficult [5,6,7].

The peak age for Addison’s disease lies between 5 and 7 years; however, cases of hypoadrenocorticism in dogs younger than 1 year have been documented [8,9,10]. In addition to blood parameters and symptoms, analyzing the size and structure of the adrenal gland is a key diagnostic factor in supporting the diagnosis of Addison’s disease [11,12,13]. Studies in adult dogs have shown that the size of the adrenal gland is reduced and its architecture altered in dogs with hypoadrenocorticism [14]. To our knowledge, the only previous study on the adrenal gland size in dogs under 1 year of age is our progenitor study in which Beagle puppies underwent consecutive ultrasound examinations of the adrenal glands between 6 and 12 months of age [15]. We observed significant growth of the adrenal glands, especially in length and caudal pole diameter.

Ultrasonographic examination is a commonly used tool to measure adrenal gland size in clinical settings [16,17]. While this method offers several advantages, including ease of implementation, the absence of general anaesthesia, low costs, and rapid results, it also has some drawbacks. The findings depend on the investigator’s experience and various factors that can influence the visibility of the adrenal glands, such as the patient’s size, body condition score (BCS), and the filling of the gastrointestinal tract [17,18].

Another limitation of ultrasonographic examinations is that this technique only provides a two-dimensional image of an organ. In contrast, computed tomography (CT) enables the reconstruction of three-dimensional images and allows for volumetric measurements of organs. Several retrospective studies in veterinary medicine have calculated adrenal gland volume from CT images of adult dogs using different software programs [18,19,20]. OsiriX^®^MD has already been used for volume analysis of the prostate, which was used by Salonen et al. [20]. This method has achieved an accuracy of ±0.8% compared to water displacement methods. To our knowledge, volumetric measurements of the adrenal glands in puppies have not previously been performed.

The aim of the study was to provide baseline data on adrenal gland volume and traditional two-dimensional measurements in puppies of twenty-two different dog breeds, and to describe volumetric measurement of adrenal glands using OsiriX^®^MD v 9.0.1 (Pixmeo SARL, 266 Rue de Bernex, CH1233 Bernex, Switzerland). We sought to determine whether there is a correlation between adrenal gland volume and age, body weight, and the patient’s adult size. Additionally, we aimed to determine whether traditional measurements of adrenal gland length, height, and width of the adrenal glands show similar trends with age, weight, and adult size compared to volumetric measurements.

## 2. Materials and Methods

### 2.1. Sample Population

In this retrospective study, patient data were collected from the data pool of the Veterinary Teaching Hospital at the Small Animal Clinic of the University of Veterinary Medicine Hannover.

The inclusion criteria consisted of pre- and post-contrast CT images of the abdomen reconstructed using a soft tissue algorithm. Additionally, only patients without any clinical or historical indications of hypoadrenocorticism, including shock, nonspecific gastrointestinal symptoms, azotemia and electrolyte abnormalities (hyponatremia, hyperkalemia) as determined by a thorough review of the medical records, were included.

CT scans that included only a pre-contrast series were considered if the image quality allowed clear visualization of the adrenal glands. Patients were 12 months old or younger. CT scans were excluded if they showed significant artefacts or if the dogs had received glucocorticoids.

### 2.2. Definition of the Groups

We categorized the patients into three age groups (0–4 months, 5–8 months, and 9–12 months) and three weight groups (0–10 kg, >10–25 kg, and >25 kg) for statistical analysis. The expected adult weights of the patients were determined using follow-up records from the clinic or breed standards derived from the official guidelines provided by the Fédération Cynologique Internationale (FCI). Mixed-breed dogs were excluded from this categorization if there was no follow-up during adulthood. Based on the adult size of the dogs, the following groups were formed: 0–10 kg (Group A), >10–25 kg (Group B), and >25 kg (Group C).

### 2.3. CT Imaging Protocols

Abdominal and full-body CT scans were conducted in sternal recumbency under general anaesthesia, adhering to the guidelines established by the Department of Anesthesiology at the Small Animal Clinic at the University of Veterinary Medicine Hannover, while taking into account the health status and pre-existing conditions of the patients.

CT examinations were performed using a detector-based spectral CT (Philips IQon Spectral CT, Philips Healthcare, Böblingen, Germany).

**Figure 1:**
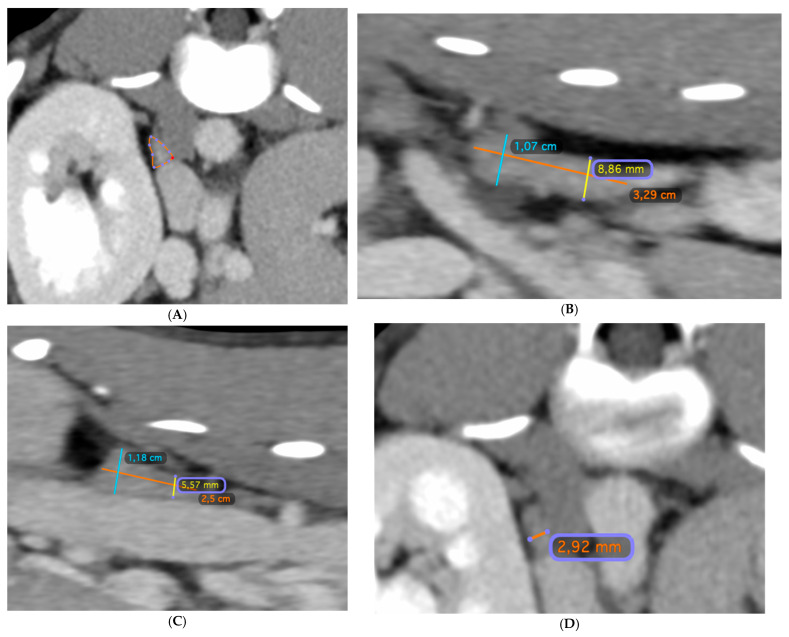
All images show adrenal glands of a 190-day-old male Irish Setter with a large intrahepatic portosystemic shunt (IHPSS). (**A**): Multiplanar reconstruction in the transverse plane showing the right adrenal gland, enclosed within a polygonal ROI. (**B**):Sagittal multiplanar reconstruction optimized for the left adrenal gland. Three linear measurements were taken: craniocaudal length (orange line), dorsoventral height of the cranial pole (blue line), and height of the caudal pole (yellow line). (**C**): Sagittal multiplanar reconstruction optimized for the right adrenal gland showing three linear measurements: craniocaudal length (orange line), dorsoventral height of the cranial pole (blue line), and height of the caudal pole (yellow line). (**D**): Transverse multiplanar reconstruction, measuring the width of the cranial pole of the right adrenal gland (orange line) adjacent to the cranial pole of the right kidney.

**Figure 2:**
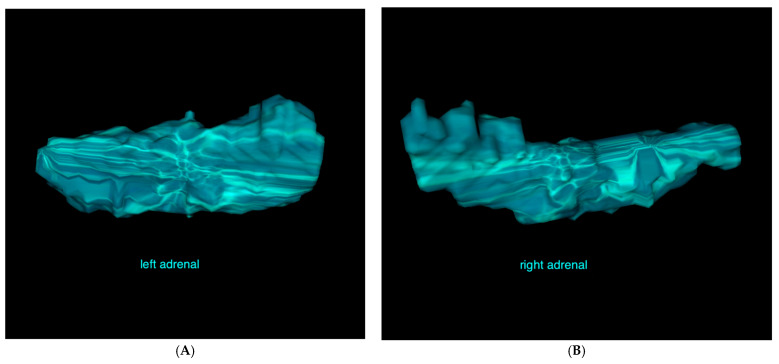
(**A**): Visualization of the volumes of the left and (**B**): right adrenal glands of a 154-day-old male Berger Blanc Suisse with neurological deficits and meningitis, derived from a series of polygonal ROIs in the transverse plane.

The following acquisition parameters were used: helical mode, maximum tube potential 120 kV, exposure 250 mAs, 1–2 mm slice thickness, 0.75 mm spacing, 512 × 512 matrix, and a medium filter algorithm. Contrast injection was performed manually with a non-ionic iodinated contrast agent (Xenetix^®^ 300, Guerbet GmbH; Sulzbach, Germany) via injecting into the vena saphena lateralis or the vena cephalica antebrachii. The images were acquired 30–60 s following bolus injection. The volumetric measurement of the adrenal glands was conducted by a board-certified radiologist (KM) using OsiriX^®^ software for three-dimensional data processing, visualization, and analysis. A closed polygon was used to outline the contour of the adrenal gland in all consecutive transverse images from the cranial to the caudal pole (Figure 1A). The software then calculated the volume of the region of interest (ROI) by multiplying the surface area by the slice thickness and subsequently summing the volumes of individual slices. The generated data were saved as a DICOM file that included both the numerical data and the visualized volumes (Figure 2).

### 2.4. CT Measurements

The CT datasets were transferred to a workstation and analyzed using commercially available DICOM imaging viewing software (OsiriX^®^ MD v 9.0.1; Pixmeo SARL, 266 Rue de Bernex, CH1233 Bernex, Switzerland). All measurements were performed by a board-certified radiologist (KM) using a soft tissue window setting.

Multiplanar reconstructions were used to determine the length (orange line, Figure 1B,C), height (blue and yellow lines, Figure 1B,C), and width (Figure 1D) of the adrenal glands. Image planes were optimized to maximize the visualization of the adrenal glands. The length of the glands, as well as the height of both the cranial and caudal poles, were measured in the sagittal plane (Figure 1B,C), while the width of the cranial and caudal poles (Figure 1D) was measured in the transverse plane. Transverse measurements were obtained at the same level where the height was measured in the sagittal plane.

Volumetric analysis of the adrenal glands was performed using the 3D rendering and analysis functions of OsiriX^®^ v.14.1.2 software. A closed polygonal region of interest was used to outline the contour of the adrenal gland in all consecutive transverse images from the cranial to the caudal pole (Figure 1D). The software automatically calculated the adrenal gland volume of the ROI by multiplying the surface area by the slice thickness and subsequently summing the volumes of individual slices. The output was saved as a DICOM file, containing both the numerical data and the corresponding 3D volume rendering (Figure 2A,B).

### 2.5. Statistical Methods

All statistical analyses were performed using commercially available software (Prism 9 by GraphPad Software, 225 Franklin Street, Fl. 26, Boston, MA, USA). Descriptive statistics are presented as mean values ± SD (range) for the continuous variables. The collected data were organized into three investigative groups based on age, weight at the time of examination, and adult size.

Initially, standard deviations and means were calculated. Following tests for normality and homogeneity (Shapiro–Wilk test), Analysis of Variance (ANOVA) tests (with Tukey’s multiple comparison test and Friedman’s test using a single pooled variance) were performed on all datasets to examine the relationship between adrenal gland size and volume and variables such as age, body weight, and expected adult size.

## 3. Results

### 3.1. Sample Population

We reviewed 543 CT cases, and 26 patients met the inclusion criteria. These patients had undergone CT examinations for suspicion of portosystemic shunts (*n* = 7), neurological disorders-central and peripheral (*n* = 6), trauma (*n* = 6), suspicion of vascular anomalies (*n* = 3), elbow joint disease (*n* = 2), angular limb deformity (*n* = 1), and ectopic ureters. (*n* = 1).

The cohort included 18 intact male and eight intact female dogs, with a median age of 192 days and an age range of 57 to 362 days.

Twenty-two different dog breeds were included: Chihuahua (*n* = 2), Golden Retriever (*n* = 2), Yorkshire Terrier (*n* = 2), Mixed Breed (*n* = 2), American Bulldog (*n* = 1), Berger Blanc Suisse (*n* = 1), Bernese Mountain Dog (*n* = 1), Boerboel (*n* = 1), Bordeaux Mastiff (*n* = 1), Boxer (*n* = 1), German Wirehaired Pointer (*n* = 1), German Longhaired Pointer (*n* = 1), German Hunting Terrier (*n* = 1), Galgo (*n* = 1), Havanese (*n* = 1), Siberian Husky (*n* = 1), Irish Setter (*n* = 1), Old English Bulldog (*n* = 1), Poodle (*n* = 1), Rhodesian Ridgeback (*n* = 1), Welsh Corgi (*n* = 1), and Miniature Dachshund (*n* = 1).

### 3.2. Groups Based on Age and Current and Adult Weight

The mean age of all patients was 198 days. The youngest dog was 57 days old, while the oldest was 363 days old. The distribution of dogs among the three age groups was as follows: 0–4 months (5), 5–8 months (14), and 9–12 months (7).

The mean weight was 14.62 kg, with the lightest dog weighing 1.04 kg and the heaviest weighing 37 kg. The weight recorded during examination was used to form the following groups: 0–10 kg (13), >10–25 kg (8), and >25 kg (5).

The adult weight of the patients was available for 22 dogs, which were re-examined at over 12 months of age in our institution. For two patients, the expected adult weight was estimated. Since two mixed-breed dogs were lost to follow-up during adulthood, we excluded them from the statistical analysis. There were eight patients in the 0–10 kg group, ten in the >10–25 kg group, and six in the >25 kg group.

### 3.3. Traditional Two-Dimensional Measurements of Length, Height and Width

**Figure 3:**
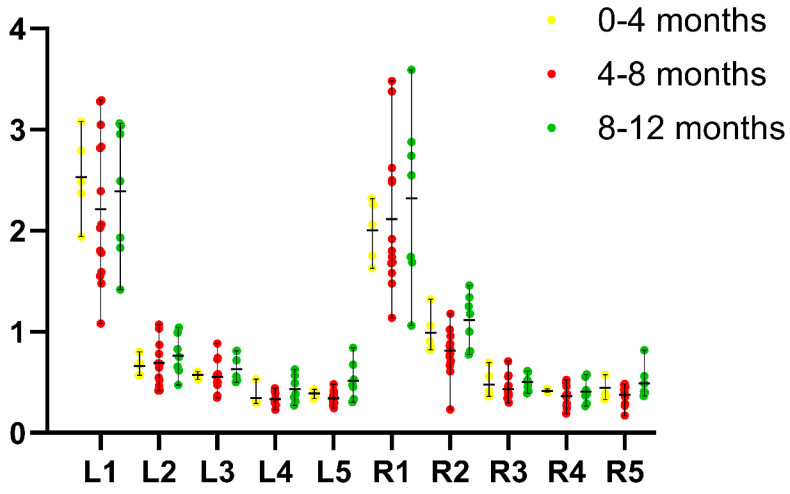
Two-dimensional measurements of the left and right adrenal glands in cm on the Y-axis; the X-axis shows the following measurements for the different age categories: L1: left adrenal gland length; L2: left adrenal gland height at cranial pole; L3: left adrenal gland height at caudal pole; L4: left adrenal gland width at cranial pole; L5: left adrenal gland width at caudal pole; R1: right adrenal gland length; R2: right adrenal gland height at cranial pole; R3: right adrenal gland height at caudal pole; R4: right adrenal gland width at cranial pole; R5: right adrenal gland width at caudal pole.

The results for length, height, and width are summarized in Table 1, and have been visualized in Figure 3.

### 3.4. Adrenal Gland Volume Analysis

Adrenal gland volumes were measured in 52 glands, including both left and right. The mean volume of the left adrenal gland was 0.51 cm (±0.33 SD), while the right adrenal gland showed a mean volume of 0.41 cm (±0.31 SD), (see Table 2).

**Figure 4:**
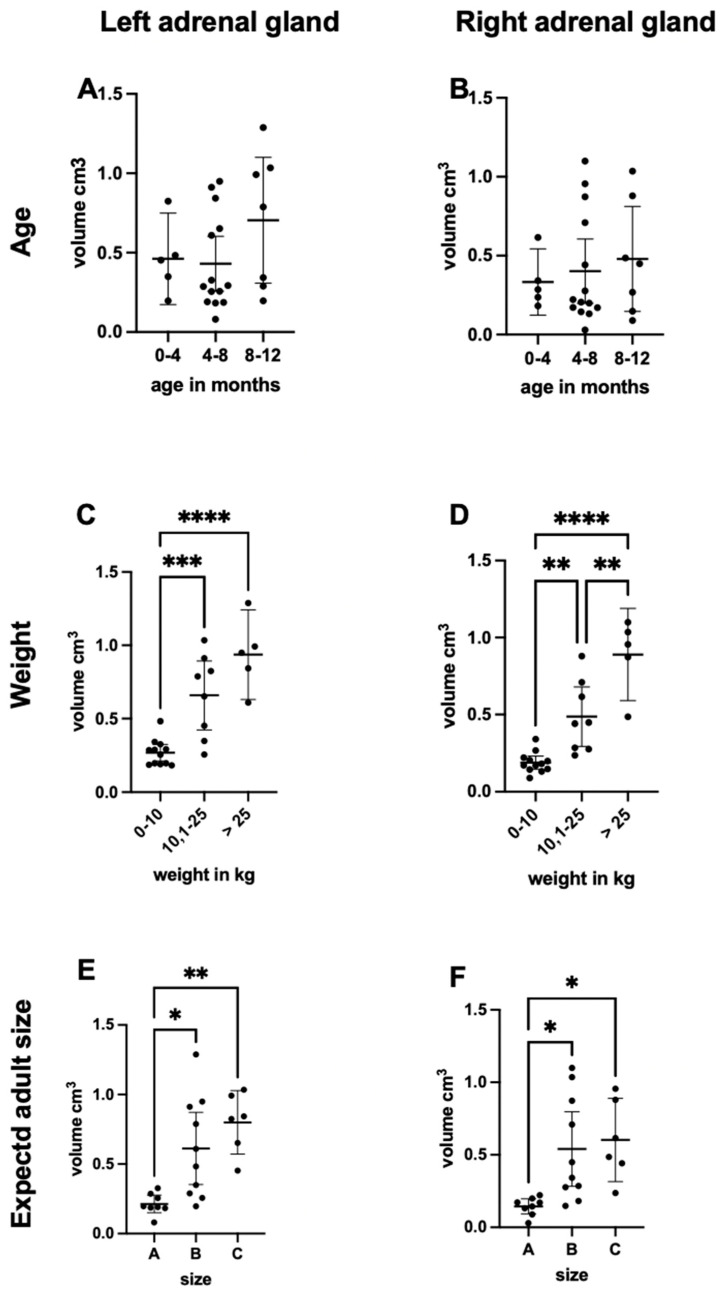
(**A**,**B**) Volume of the left and right adrenal glands in relation to age in months. (**C**,**D**) Volume of the left and right adrenal glands in relation to weight in kg. (**E**,**F**) Volume of the left and right adrenal glands in relation to adult size ((A): small, (B): medium, (C): large). Statistical significance of post hoc test (Turkey in the case of ANOVA and Dunn’s in the case of the Friedman test) is indicated by * (*p* < 0.05), ** (*p* < 0.01), *** (*p* < 0.001) and **** (*p* < 0.0001).

In the left adrenal gland, the mean volume was 0.46 cm^3^ (±0.21 SD) in the 0–4-month age group, 0.43 cm^3^ (±0.29 SD) in the 4–8-month group, and 0.70 cm^3^ (±0.40 SD) in the 8–12-month group (Figure 4A).

In the right gland, we observed a consistent increase in adrenal volume, which rose from 0.33 cm^3^ (±0.15 SD) to 0.40 cm^3^ (±0.34 SD), reaching a peak of 0.48 cm^3^ (±0.33 SD) in the oldest group (Figure 4B). There was no statistical significance between the values of all three age groups.

Adrenal gland volume correlated positively with body weight (Figure 4C,D).

In dogs weighing 0–10 kg, the left and right adrenal glands averaged 0.25 cm^3^ (±0.10 SD) and 0.18 cm^3^ (±0.07 SD), respectively.

In the >10–25 kg group, volumes increased to 0.66 cm^3^ for the left (±0.26 SD) and 0.49 cm^3^ for the right adrenal gland (±0.21 SD).

Dogs weighing more than 25 kg exhibited the largest adrenal glands with a left mean 0.94 cm^3^ (±0.22 SD), right mean 0.89 cm^3^ (±0.22 SD) see (Table 2). There was a statistically significant difference between the values from the lightest and the heaviest groups (*p* < 0.0001), on both sides. On the right side, differences between all weight categories (0–10 kg and the >10–25 kg: *p* < 0.01; >10–25 kg and >25 kg: *p* < 0.01), and on the left side, the ones from the 0–10 kg and the >10–25 kg groups were statistically significant (*p* < 0.001).

Adrenal gland size was also affected by adult weight (Figure 4E,F).

Group A (small breeds) showed mean gland volume 0.21 cm^3^ (±0.07 SD), on the left and 0.14 cm^3^ (±0.05 SD)on the right side. Group B (medium-sized breeds) showed mean gland volume 0.61 cm^3^ (±0.34 SD), on the left and 0.54 cm^3^ (±0.34 SD) on the right side.

Group C (large breeds) had the highest values for mean gland volume 0.80 cm^3^ (±0.20 SD), on the left and 0.60 cm^3^ (±0.25 SD) on the right side. There was a statistically significant difference between the measurements for the small and medium-sized groups (*p* < 0.05), on both sides. The values from the small and large adult size groups also showed statistically significant differences for both adrenal glands (left: *p* < 0.01; right: *p* < 0.05).

The largest volume for the left adrenal gland was 1.29 cm^3^, observed in a German Wirehaired Pointer that belonged to the oldest group (331 days) and the >25 kg group, but was classified in group B regarding adult size. The maximum volume of the right adrenal gland was 1.10 cm^3^, recorded in an American Bulldog in the >25 kg group, which was also classified in group B with respect to adult size and belonged to the middle age group (230 days).

## 4. Discussion

Our study provides baseline data on adrenal gland volume and traditional two-dimensional measurements in puppies. The described volumetric assessments of adrenal glands in dogs under 1 year of age can be easily performed by a radiologist experienced with OsiriX^®^MD v 9.0.1. Although this method is not suitable for routine clinical use due to the extra time required, it may serve as a valuable diagnostic tool when a more detailed evaluation of the adrenal glands is necessary.

Overall, adrenal gland volumes did not differ significantly across age groups, despite the observed trends. Previous research has shown that significant adrenal growth progresses with both age and body weight [17,21,22]. In our study, we could not prove significant growth due to the small study population and the absence of follow-up measurements during adulthood.

Nevertheless, the group of 8–12-month-old dogs in our study showed the highest volumes within the sample. Notably, the mean values exhibited a mild but consistent increase in the volume of the right adrenal gland with advancing age, supporting the hypothesis that the adrenal glands continue to grow as an animal matures. Similarly, the left adrenal gland also demonstrates an increase in volume with age, although we observed a mild regression in volume in the 4–8-month group. Differences in growth rates or nonlinear development between the left and right adrenal glands might reflect anatomical or physiological variations or differing endocrine responses [21,23,24,25]. It is also essential to recognize that the adrenal glands produce not only glucocorticoids and mineralocorticoids but also sex hormones, suggesting that changes in adrenal gland size or volume could occur during puberty [19,23,25,26]. The relationship between gland size and sexual maturity warrants further investigation in future studies. Prospective hormone measurements taken during imaging and follow-up would provide valuable insights, but were beyond the scope of this retrospective study.

In our progenitor study, in which Beagle puppies underwent consecutive ultrasound examinations of the adrenal glands between 6 and 12 months of age, we observed significant growth of the adrenal glands, particularly in length and caudal pole diameter [15]. Our mean adrenal volumes in this study were slightly lower than those reported for adult dogs by Bertolini et al. [18] and Büttelmann et al. [19], indicating that adrenal growth may continue beyond 12 months.

We observed a significant positive correlation between adrenal gland volume and body weight, consistent with the veterinary literature on adrenal gland volume, length, and caudal pole thickness [20]. The most notable effect was seen with current body weight, with heavier animals having significantly larger volumes in both the left and right adrenal glands. This highlights the importance of considering body weight when assessing adrenal size in young dogs and may help refine diagnostic criteria through weight-adjusted reference values.

The adrenal gland volumes in dogs weighing >10–25 kg (0.66 cm^3^ left, 0.49 cm^3^ right) align with Büttelmann et al., confirming a positive correlation between adrenal gland size and body weight, with the largest volumes in dogs from the highest weight category (>25 kg). Especially in clinical assessments for disorders such as hypoadrenocorticism, in which adrenal gland atrophy is a key diagnostic feature, accurate evaluation of gland size must include weight-specific reference ranges to prevent misinterpretation [15,19,24,27].

Adrenal gland volumes showed a clear, significant connection with the dogs adult body weight. Larger breeds consistently exhibited larger adrenal glands than smaller breeds, possibly due to increased metabolic demands [16,17,19,28]. The two largest adrenal glands in our study belonged to dogs from the highest weight group, although both were in group B (>10–25 kg) based on their adult size. Although adult body weight was significantly correlated with adrenal gland volume, our findings suggest that the current body weight at the time of examination has a more substantial impact on adrenal volume than adult size.

The range of traditional measurements of the length and width of the cranial and caudal poles of the adrenal glands was broader in our study, with higher maximum values, compared to the progenitor study with Beagle puppies [15]. This supports our finding that adrenal gland size depends on weight, as the Beagles were within the lower or medium weight groups. The highest values for the traditional measurements in our study were observed in dogs in the medium or highest weight groups, which explains the wider range and emphasizes the need for weight-adjusted reference values for puppies.

The traditional measurements of length, height, and width showed notable variation within the studied canine population. We observed consistent asymmetry, with the left gland generally longer, matching ultrasonographic data from previous research [24].

Notably, the length of the left adrenal gland showed nonlinear values: initially high in the 0–4-month age group, slightly decreasing during the 4–8-month interval, and then increasing again in the 8–12-month group. This observation could reflect adrenal cortex development [21,26,29]. In contrast, the right adrenal gland exhibited a steady increase in length with age, suggesting differing developmental dynamics between the two glands. These findings align well with the sonographic results of our progenitor study, which described mild differences in growth patterns between the left and right adrenal glands, with the right side being consistently larger in volume and showing more uniform growth [15].

Melián et al. reported upper size ranges for the adrenal glands based on body weight: 0.51–0.73 cm (right) and 0.53–0.87 cm (left). In our study, the right adrenal glands did not exceed this range, but one left adrenal gland was slightly above the reference at 0.89 cm. This outlier was from a 190-day-old male Irish Setter, a medium-sized breed. Unfortunately, pathological enlargement cannot be excluded retrospectively, but it is considered less likely given the divergence of only 0.02 cm.

Our findings support CT as a reliable tool for assessing adrenal gland size, especially in young puppies with limited cooperation during ultrasound examinations. Ultrasound imaging of the adrenal glands remains highly reliant on the skills of the ultrasonographer, whereas CT interpretation is more objective. However, ultrasound is typically the first imaging modality chosen due to its greater accessibility, cost-effectiveness, and the fact that sedation is often not needed, which mirrors the drawbacks of CT. These two modalities are therefore used together, with CT being the preferred method mainly in larger patients and those requiring a more objective assessment of adrenal gland size.

Our small sample size restricted the statistical power. Studies with a larger sample should include the BCS to better understand the effects of body composition [30,31].

We understand that adult size is a factor assessed in most patients, but it was estimated in two, which introduces some potential inaccuracy. Nevertheless, we found it interesting to consider the impact of expected adult size to enhance our understanding of adrenal sizes in puppies.

Another limitation is the lack of information concerning thyroid hormone levels and baseline cortisol levels in the dogs. These could provide valuable insights into metabolic regulation, potentially influencing adrenal gland function. Future prospective studies should include measurements of baseline cortisol or even ACTH-stimulation tests to thoroughly investigate possible links between adrenal gland morphology and to definitively exclude patients with adrenal gland diseases.

The lack of a gold standard method for validating the volumetric measurements of the adrenal glands highlights another limitation of our study. Techniques such as water displacement volumetry, which could offer an objective and highly accurate reference for organ volume, were unavailable for this study. Without such a reference, we cannot definitively confirm the accuracy of our volumetric assessments derived from imaging data.

## 5. Conclusions

In conclusion, our data offer baseline information on adrenal gland volume and size parameters in dogs under 1 year of age. The primary factor influencing adrenal gland volume was body weight. A moderate effect was seen for the expected adult size, while the age of the patient showed no apparent influence. We found that volumetric measurements of adrenal glands in puppies using CT were feasible and may provide valuable information when screening for adrenal pathologies. Further research with larger sample sizes and a prospective design, including consideration of the body condition score, would help to refine diagnostic criteria for adrenal gland-related diseases in veterinary medicine.

## Figures and Tables

**Table 1:** Two-dimensional adrenal gland measurements (length, height, and width).

Measurements	Left (Mean ± SD, cm)	Right (Mean ± SD, cm)
length	2.32 (±0.64)	2.15 (±0.68)
Cranial pole height	0.71 (±0.19)	0.93 (±0.26)
Caudal pole height	0.58 (±0.13)	0.49 (±0.11)
Cranial pole width	0.36 (±0.10)	0.39 (±0.10)
Caudal pole width	0.40 (±0.13)	0.42 (±0.12)

**Table 2:** Volumetric measurements of the left and right adrenal gland.

Age (Days)	Breed	Sex	Body Weight (kg)	Adult Weight Category	Left Adrenal Gland Volume (cm^3^)	Right Adrenal Gland Volume (cm^3^)
57	German Longhaired Pointer	f	5.0	B	0.20	0.18
87	Rhodesian Ridgeback	m	13.4	C	0.45	0.24
110	Husky	f	10.2	B	0.35	0.29
118	Boerboel	m	24.0	C	0.82	0.62
122	Golden Retriever	m	9.4	B	0.48	0.34
136	Yorkshire Terrier	f	2.0	A	0.19	0.13
152	Boxer	m	16.2	B	0.26	0.28
154	Berger Blanc Suisse	m	24.4	C	0.65	0.44
169	Chihuahua	m	1.04	A	0.08	0.03
171	Yorkshire Terrier	m	2.2	A	0.19	0.17
173	Welsh Corgi	m	5.8	A	0.29	0.22
190	Irish Setter	m	15.6	B	0.91	0.71
194	Havanese	f	2.4	A	0.18	0.14
209	Bordeaux Mastiff	m	43.0	C	0.84	0.96
220	Miniature Dachshund	m	4.2	A	0.26	0.17
230	Golden Retriever	m	31.6	B	0.61	0.87
230	American Bulldog	m	32.1	B	0.95	1.10
246	German Hunting Terrier	f	5.8	A	0.33	0.20
253	Galgo	m	21.0	C	1.03	0.88
255	Old English Bulldog	m	20.0	B	0.79	0.45
272	Bernese Mountain Dog	m	37.0	C	0.65	0.44
274	Poodle	m	6.4	B	0.29	0.15
331	German Wirehaired Pointer	m	33.6	B	1.29	1.04
362	Chihuahua	f	1.8	A	0.20	0.09
155	Mixed Breed	f	6.8	Not categorized	0.29	0.21
289	Mixed Breed	f	5.2	Not categorized	0.34	0.27

Age in days, breed, sex (f = female, m = male), body weight (kg), adult weight category (A = 0–10 kg, B = >10–25 kg, C = >25 kg), and volumetric measurements of the left and right adrenal glands (cm^3^).

## Data Availability

The original contributions presented in this study are included in the article. Further inquiries can be directed to the corresponding authors.

## Abbreviations

abbrevation	meaning
ACTH	Adrenocorticotropic Hormone
BCS	Body Condition Score
CT	Computed Tomography
DD	Differential Diagnosis
DICOM	Digital Imaging and Communications in Medicine
F	Female
FCI	Fédération Cynologique Internationale
HU	Hounsfield Units
i.m.	Intramuscular
i.v.	Intravenous
KM	Kristina Merhof
L1	left adrenal gland length
L2	left adrenal gland height cranial pole
L3	left adrenal gland height caudal pole
L4	left adrenal gland width cranial pole
L5	left adrenal gland width caudal pole
R1	right adrenal gland length
R2	right adrenal gland height cranial pole
R3	right adrenal gland height caudal pole
R4	right adrenal gland width cranial pole
R5	right adrenal gland width caudal pole
M	Male
MRI	Magnetic Resonance Imaging
PRAA	Persistent Right Aortic Arch
p.o.	Per os (oral administration)
ROI	Region of Interest
SD	Standard Deviation
SPEC	Spectral Computed Tomography
Th	Thoracic vertebra
UKC	United Kennel Club
WC	Weight Category
OsiriX^®^MD	medical image processing software developed by Pixmeo SARL
